# Are there rapid feedback effects on Approximate Number System acuity?

**DOI:** 10.3389/fnhum.2013.00270

**Published:** 2013-06-12

**Authors:** Marcus Lindskog, Anders Winman, Peter Juslin

**Affiliations:** Department of Psychology, Uppsala UniversityUppsala, Sweden

**Keywords:** Approximate Number System, feedback, learning, numerosity, numerical, estimation

## Abstract

Humans are believed to be equipped with an Approximate Number System (ANS) that supports non-symbolic representations of numerical magnitude. Correlations between individual measures of the precision of the ANS and mathematical ability have raised the question of whether the precision can be improved by feedback training. A study (DeWind and Brannon, 2012) reported improvement in discrimination precision occurring within 600–700 trials of feedback, suggesting ANS malleability with rapidly improving acuity in response to feedback. We tried to replicate the rapid improvement in a control group design, while controlling for the use of perceptual cues. The results indicate no learning effects, but a minor constant advantage for the feedback group. The measures of motivation suggest that feedback has a positive effect on motivation and that the difference in discrimination is due to the greater motivation of participants with feedback. These results suggest that at least for adults the number sense may not respond to feedback in the short-term.

## Introduction

Non-symbolic representation of numerical magnitudes is an ability shared by human adults, infants, and non-human animals (Feigenson et al., 2004). The Approximate Number System (ANS) claimed to support this ability is thought to represent numbers in an analog and approximate fashion with increasingly imprecise representations with increasing numerosity (Dehaene, 2008; but see, Brannon et al., 2001). The acuity of the ANS, conceptualized as the smallest numerical change that can be reliably detected, is often quantified by a Weber fraction (*w*). Recent studies show that there is a substantial individual variability in acuity of the ANS (*w*:s for adults often lie in the range 0.1–0.45, see e.g., Pica et al., 2004; Halberda and Feigenson, 2008; Halberda et al., 2008; Tokita and Ishiguchi, 2010), and that the acuity improves from childhood to adolescence (Halberda and Feigenson, 2008).

Brain-imaging studies have indicated that the ANS has a neurological basis in the intraparietal sulcus (IPS) on the lateral surface of the parietal lobe (Castelli et al., 2006; Piazza et al., 2006) and studies on macaque monkeys have even found specialized neurons (numerons) within the IPS that are sensitive to numerosity (Nieder et al., 2002). The IPS is activated when people attend to or compare the number of objects in a set, when they observe numbers in different modalities, and when they perform simple arithmetic tasks (Piazza et al., 2004; Piazza and Izard, 2009) suggesting that the ANS is supramodal and independent of perceptual variables. Further support for this was found in a recent study using single-cell recordings in the primate brain (Nieder, 2012a). It was shown that there are neurons that encode numerosity irrespective of stimulus modality (visual/auditive) (see Nieder, 2012b for a review of the physiology of “number neurons”).

Halberda et al. (2008) demonstrated that a relationship between ANS acuity and formal mathematics achievement exists when controlling for a set of cognitive abilities. Further, children with dyscalculia have been shown to suffer from an ANS-impairment (Piazza et al., 2010; Mazzocco et al., 2011) highlighting the question of whether it is possible to improve the acuity of the ANS with training. It is possible that the association between the precision of the ANS and mathematical ability exists because the ANS lays the foundation for higher-level arithmetical concepts. This remains yet to be shown, but the finding that it is possible to improve the functioning of this system would have tremendous applied and clinical impact should such a causal link be established.

### Measuring training effects on ANS acuity

If empirical studies show that participants improve in their discrimination of numerosities after presentation of feedback, this effect could in principle be due to at least four different and independent explanations (or a combination of any of these): (1) the improvement (e.g., in terms of lower *w*:s) may be due to practice in regard to more superficial attentional and procedural aspects of the task, such as learning how to best attend to the stimuli, to decrease error when responding, etc.; (2) the feedback may have a motivational effect on the participants' performance, making them try harder. These first two explanations can be regarded as relatively superficial in the sense that they are not related intrinsically to numerosity discrimination, but apply, potentially, to any task content; (3) the participants may improve or shift their strategies in the use of indirect perceptual cues for numerosity, so that they use them more efficiently. They might, for example, learn to weight the cues in a more optimal way to arrive at more accurate estimates of numerosity. The ANS, as described above, involves an abstract representation independent of perceptual variables. However, several settings have shown the interference of lower level perceptual variables such as element-size or density (Tokita and Ishiguchi, 2010) and there are accounts that reject the idea of an ANS altogether on these grounds, proposing that the judgments stem from weighting of multiple visual cues (Gebuis and Reynvoet, 2012); (4) the feedback may truly “sharpen” an abstract but malleable ANS, improving its acuity by experience-dependent functional plasticity through enhanced selectivity in neurons that represent numerosity. Most studies on training of the ANS have had the aim to demonstrate the last of these four explanations. The unambiguous demonstration of a sharpening of an abstract ANS, while excluding the other three possible explanations, would indeed be a highly interesting observation from a psychological perspective.

To exclude explanation 1 above, a control group that performs the task without feedback is needed^1^. Surprisingly, there seem to be no studies that have used a control group as a comparison. A motivational explanation may be probed for by obtaining self-ratings of motivation. Spontaneous verbal reports from pilot participants have indicated that a number of them found the task much more interesting and engaging when receiving feedback (see e.g., Kluger and DeNisi, 1996, for a review of how feedback influences motivation). No previous studies have investigated the motivational effects on numerosity discrimination or controlled for the motivational effects of introducing feedback in such discrimination tasks.

The participants may use perceptual cues rather than numerosity to solve non-symbolic discrimination tasks, and it may be practically impossible to completely rule out the possibility that they fully or partially do so (Gebuis and Reynvoet, 2012). However, any study aiming to make the claim that the ANS is highly malleable (when the ANS is considered a fundamentally abstract representation independent of perceptual variables), can make a strong case for this conclusion only if the stimuli are arranged so as not to promote the use of the salient perceptual cues and if it can be convincingly demonstrated that the positive effect of feedback is not mediated by a shift in the use of these salient perceptual cues. This means that the performance should improve for all stimuli, irrespective of perceptual arrangement.

In sum: regardless of whether numerosity discrimination involves an ANS or the weighting of indirect perceptual cues, a positive training effect on numerosity discrimination is primarily of interest if the two first explanations are controlled for (being familiarized with the attentional and procedural demands of the task or by increased motivation). If one, in addition, wants to claim an improvement in the acuity of an abstract ANS, one needs to try to rule out in a convincing way, that the effect is mediated by a shift in the use of perceptual cues.

### Empirical findings

Results from studies of training on children (e.g., Wilson et al., 2006a,b, 2009) have been mixed and the lack of control groups makes it hard to separate the effects on ANS per se from the other explanations discussed above. Two recent studies on adults (Tokita and Ishiguchi, 2010; DeWind and Brannon, 2012) have shown effects of feedback on performance in numerosity judgment tasks. Tokita and Ishiguchi (2010) manipulated perceptual cues (element size and array area) in a comparative numerosity judgment task and investigated effects of feedback on the influence of these cues on judgments. They showed that people initially responded to the perceptual cues but could learn not to attend to them, with lower *w*:s as a result. They concluded that observers could learn to suppress the interference of perceptual cues or the influence of prior knowledge with practice under feedback. DeWind and Brannon (2012) provided participants with trial-by-trial feedback on six one hour sessions and found immediate effects in terms of improved (lower) *w*:s that appeared in the first session and then remained constant.

Both of the studies above show improvement in numerosity judgments following feedback, but different claims were made by the authors. The purpose of the Tokita and Ishiguchi (2010) study was to examine whether people can learn not to attend to perceptual cues that were deliberately introduced in the stimuli. The authors did not claim that their findings show a sharpening of the ANS. DeWind and Brannon (2012), however, made the stronger claim that the ANS is malleable and its acuity rapidly improved in response to feedback, conveying a positive message about the possibility of rapid enhancement of the ANS with feedback. The interpretation of the results is not clear-cut, however.

First, the study lacks a control group, leaving the possibility that the observed improvement may be due to superficial practice effects rather than a true effect of malleability of the ANS. Second, the presence of strong perceptual cue reliance in the study may be problematic. To control for perceptual cues DeWind and Brannon used three types of stimuli. The cumulative area of the dots was either larger for the more numerous set of dots (congruent), smaller for the more numerous set (incongruent) or constant for both sets. This arrangement of stimuli has the unfortunate consequence of confounding perceptual variables with numerosity in that the less numerous sets have a larger dot-size on 2/3 of the trials. The results indeed indicated that the overall use of cumulative area as a cue to number decreased and that this decrease was parallel to the improvement of *w*:s.

It was shown that whereas participants in the first session performed better on congruent trials, by the last session performance was better on incongruent trials, an effect that was statistically significant when excluding outliers. Thus, participants seem to have shifted from one perceptual strategy (cumulative area) to another (size). When analyzed separately, performance did indeed improve for trials where cumulative area was held constant. However, as suggested by the results for congruent and incongruent trials, it could be that participants relied on dot-size rather than cumulative area. This interpretation is reasonable, because, again, dot-size was confounded with numerosity and previous research indicates that people are influenced by dot-size when judging numerosity (Krueger, 1984; Vos et al., 1988; Shuman and Spelke, 2006). To substantiate the suspicion that perceptual cues were a significant factor in the above study we performed additional analyses on the data from the study^2^. The malleability hypothesis as an explanation of the data in terms of an improvement in a perceptual independent number system implies that participants generally should improve in their performance for all items, irrespective of the perceptual constellation. If participants on the other hand improved because they learned to pick up the perceptual cues, we would expect a different pattern for different stimuli. More specifically, if participants picked up the confound of dot-size and numerosity and responded to this, we would, depending on the degree to which they rely on these cues expect impaired improvement, or even a decrease in performance for congruent trials, for which this cue is misleading. This stimulus set was never analyzed separately in the original study. An analysis of performance proportion correct (PC) on congruent trials indeed shows that not only does performance fail to improve, but actually quite drastically drops for these stimuli, from 0.79 in the pre-test to 0.69 in the post-test. The deterioration is almost twice as large as the overall improvement reported by DeWind and Brannon with all stimuli included, and highly statistically significant [*F*_(1, 18)_ = 10.3, *p* < 0.005]. Thus, 4 days of training with feedback has the effect of making participants worse at discriminating between numerosities when the more numerous set has a larger dot-size than the less numerous. This analysis thus substantiates the suspicion that perceptual cues are a key factor behind the results of DeWind and Brannon.

To exclude the explanation that participants shift their strategies in attending to perceptual cues, learning need to be demonstrated in a setting where the stimuli is arranged so as not to promote the use of perceptual cues and where it can be demonstrated that the participants do not, at least primarily, rely on the salient perceptual cues.

We use a numerosity task closely based on the one designed by Halberda and colleagues (2006, 2008; see the method section below) which controls for cumulative area on half the trials and average dot-size on the other half of the trials. (With spatially overlapping sets and random dot placement, area-controlled trials also control for other continuous perceptual variables such as total dot density, inter-dot distance and total envelope set size.) With this procedure, several studies (see e.g., Cantlon and Brannon, 2006, 2007; Halberda and Feigenson, 2008; Halberda et al., 2006, 2008; Mazzocco et al., 2011) have suggested that participants do rely on numerosity rather than on perceptual cues. This has also been confirmed on a large number of participants in our lab. This procedure has two benefits with respect to the evaluation of explanation three as discussed above. First, because area and size controlled trials are presented intermixed in an individually randomized order, the task does not invite the use of perceptual cues. Second, if participants nevertheless use perceptual cues instead of numerosity, and change in the way they use such cues when feedback is introduced, it will be possible to evaluate this in post hoc statistical tests.

In sum: Tokita and Ishiguchi (2010) demonstrated that the participants can learn from feedback to suppress perceptual cues. There are indications of ANS malleability in the results in DeWind and Brannon (2012), but it remains unclear if these effects are explained by more superficial practice effects, perceptual strategies or effects on motivation of introducing feedback.

### The present study

The purpose of the present study accordingly was to replicate the rapid learning reported by DeWind and Brannon (2012) in a control group design and with a task that does not promote use of perceptual cues, and where it can be detected if a positive training effect is mediated by a shift in the use of these perceptual cues. Motivational factors will be controlled for by obtaining self-rated motivation scores.

ANS malleability predicts a pattern of data with a gradual improvement in the numerosity discriminations in the experimental group accompanied by a lack of improvement in the control group. Superficial practice effect predicts a pattern of data where both the control and experimental group improve to a similar degree. A motivational effect of feedback predicts that the experimental group will perform at higher levels than the control group once that feedback is provided, presumably with an abrupt improvement in the performance when the feedback is introduced, rather than as an incremental function. The finding that effects remain when, after a training period feedback is suddenly withheld would not exclude this possibility. It could be, for example, that the withdrawal of feedback itself signals that a final test of learning will take place that sustains high motivation per se. The perceptual cues will be probed for by undertaking separate analyses for size-controlled vs. area-controlled stimulus presentations. If the participants learn to pick-up these perceptual cues, we expect a pattern of results where the differences in the performance on these stimulus types turn up in the experimental group, but not in the control group.

The performance in number discrimination tasks might be related to a more general ability to process visual stimuli rapidly, so called mental speed (Deary and Stough, 1996). To investigate the relationship between ANS acuity and mental speed we therefore included an inspection time test. The ANS is considered a fundamental core cognitive ability with modular properties and should thereby possibly be inaccessible to meta-cognitive monitoring and conscious thought (Mandelbaum, 2013). While there are several types of meta-cognitive monitoring (Merkle and Weber, 2011) we were interested in participants' ability to compare their own performance with the performance of others. Therefore, the participants were asked to rate their own performance in the ANS and inspection time tasks relative to the other participants in the study.

We focus on the region of feedback trials where DeWind and Brannon (2012) observed the effects (these occurred after 648 trials, with no further improvement of an extra 2500 trials)^3^. We used the same exposure time, the same type of stimuli, the same (spatially intermixed) presentation method, similar stimulus ratios and a sample of participants of age and background comparable to DeWind and Brannon (2012)^4^. Thus, in all important aspects the present study is a replication of the DeWind and Brannon (2012) study but with alterations to control for practice effects, reliance on perceptual cues, and motivational effects.

## Method

Participants (9 Male, 31 Female) were undergraduate students from Uppsala University with a mean age of 25.4 years (*SD* = 5.7)^5^. All participants gave informed consent to participate.

The task, based on Halberda et al. (2008), had a pre-test, a training block, and a post-test consisting of 200, 1000, and 100 trials, respectively. On each trial, participants saw spatially intermixed blue and yellow dots on a monitor. Exposure time (200 ms) was too short for the dots to be serially counted. We used five ratios between the two sets of dots (1:2, 3:4, 5:6, 7:8, 9:10) with the total number of dots varying between 11 and 30. One 5th of the trials consisted of each ratio. Half of the trials had blue and half had yellow as the more numerous set. The dots varied randomly in size. To counteract the use of perceptual cues we matched dot arrays either for total area or for average dot-size. The participants judged which set was more numerous by pressing a color-coded keyboard button.

In training, participants (*n* = 20) in the feedback condition received immediate “correct/wrong” feedback on their judgment while participants (*n* = 20) in the control condition did not. The feedback was presented with the words “correct” and “wrong,” written in green and red respectively, in close proximity to the dot stimulus. Both groups were instructed to try to improve, and achieve as many correct answers as possible. Participants carried out all 1300 trials of the experiment in a single session, lasting approximately 120 min, with the possibility to take breaks after each block of 200 trials.

After the post-test block, participants rated their own motivation during the test on a 1 (very low motivation) to 5 (very high motivation) scale and performed a visual inspection time task, based on Deary et al. (2004) that measures perceptual speed. The inspection time task is a two-alternative forced-choice visual backward masked task in which the participants are exposed to two parallel lines of different lengths, the ratio of which is held constant for all trials, and are to decide which is longer. The line stimulus are presented with one of five presentation times (25, 40, 60, 80, 100 ms) with one 5th of the trials from each presentation time. Participants rated their own performance relative to the other participants by estimating the percentage of participants with a lower percentage correct than themselves for both the ANS acuity task and the inspection time task (i.e., their percentile rank).

### Modeling of ANS acuity

We used a classical psychophysics model that relies on a linear form of the ANS, to model performance in the ANS acuity task. Earlier work (e.g., Halberda et al., 2008) has shown this to be a plausible model of performance in numerical discrimination tasks. Percentage correct was modeled as a function of increasing ratio between the two sets of blue and yellow dots [larger sample (*n*_1_)/smaller sample (*n*_2_)]. The two sets are represented as Gaussian random variables with means *n*_1_ and *n*_2_ and standard deviations *w* · *n*_1_ and *w* · *n*_2_, respectively. Subtracting the Gaussian for the smaller set from that for the larger set returns a new Gaussian with mean *n*_2_ − *n*_1_ and standard deviation $w\sqrt{n_{1}^{2}+n_{2}^{2}}$. Percentage correct is then equal to 1—error rate, where error rate is defined as the area under the tail of the resulting normal curve computed as:

(1) $$\frac{1}{2}erfc\left(\frac{\left|n_{1}-n_{2}\right|}{\sqrt{2w}\sqrt{n_{1}^{2}+n_{2}^{2}}}\right),$$

where *erfc* is the complementary error function. This fits percentage correct in the ANS acuity task as a function of the Gaussian approximate number representation for the two sets of dots with *w* as a single free parameter. The individual *w* obtained describes the standard deviations for the Gaussian representation of the ANS acuity, describing how much the two Gaussian representations overlap and predicting an individual's percentage correct on a numerical discrimination task. We used this model to find the best fit for each individual for the pre-test, training, post-test blocks, thus obtaining individual *w*:s.

## Results

**Figure 1:**
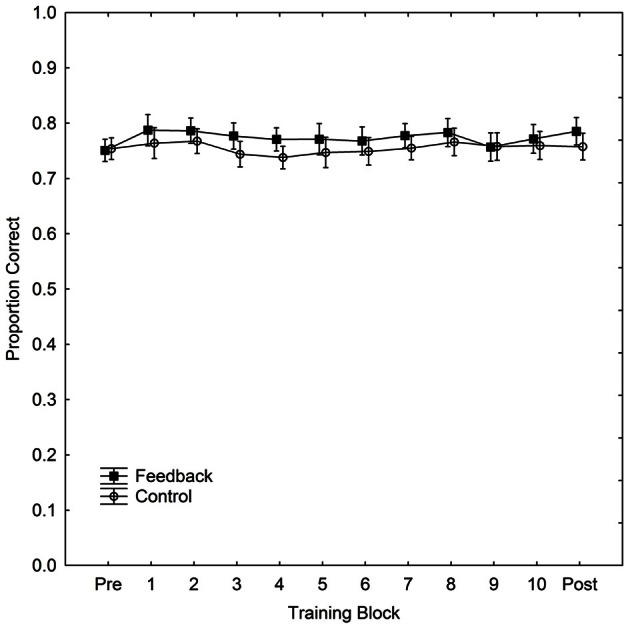
**Performance (proportion correct) in the ANS acuity task as a function of training block and condition during 1000 trials of training.** The figure also includes performance in pre- and post-tests. Vertical bars denote 95% confidence intervals.

Performance, for both *w* and PC, for each part of the experiment (pre-test, training, and post-test) and for the control and feedback conditions separately is summarized in Table 1. The table also includes *p*-values for tests of the difference (independent *t*-tests) in performance between the two groups for each part respectively. (The effect of feedback, in terms of Cohen's *d* for the difference in PC reported in the DeWind and Brannon (2012) between performance in the control and at training session 1 was 1.21. With our between subjects design, an alpha-level of 0.05 and a two-tailed test (i.e., those reported in Table 1), we have a power of 0.98 to detect an effect of the corresponding size. Thus, the present study has a very high power of detecting an effect at least as large as that previously observed.) Performance was marginally better in the feedback than in the control group in all parts except the pre-test, but there were no signs of learning during the training. The improvement in the feedback group occurred immediately when feedback appeared. Figure 1 shows the data from the training block separated into 10 sub-blocks of 100 trials, together with the data from pre- and post-tests, in order to examine the possibility of a modest initial learning effect that quickly levels out. PC, rather than *w*, is used in the figure to depict performance.

**Table 1:** **Performance, both as *w* and PC, in each part of the experiment (pre-test, training, post-test) and the P_cue_ index for the control and feedback conditions respectively**.

		**Condition**				
		**Control**		**Feedback**		
**Block**	**Measure**	***M***	***SD***	***M***	***SD***	***p*-value^a^**
Pre	PC	0.75	0.05	0.75	0.04	0.83
	*w*	0.24	0.05	0.24	0.07	0.80
	P_cue_	0.00	0.06	−0.01	0.07	0.40
Training	PC	0.75	0.03	0.77	0.04	0.07
	*w*	0.23	0.05	0.21	0.04	0.12
	P_cue_	0.01	0.05	0.01	0.03	0.68
Post	PC	0.76	0.06	0.79	0.05	0.11
	*w*	0.24	0.10	0.19	0.06	0.08
	P_cue_	0.00	0.12	−0.01	0.07	0.60

a *p-values are for independent t-tests for the difference between the two groups for each performance measure in each part of the experiment (A positive score for the P_cue_ index indicates superior performance on area-controlled stimuli)*.

It is clear that there is no learning in either group, but that the feedback group has a slight advantage already at the moment when feedback is introduced. If anything, the figure indicates that the difference in PC between the two groups decreases with training. We calculated PC scores and *w* for each participant for each training block of 100 trials. We then fitted a regression model to individual PC scores and *w* with regressors for training block number and participant. We found that participants did not improve with training in neither the feedback condition (PC: *b* = −0.002, *p* = 0.19, *R*^2^ = 0.05; *w*: *b* = 0.002, *p* = 0.29, *R*^2^ = 0.05), nor in the control condition (PC: *b* = 0.0003, *p* = 0.78, *R*^2^ = 0.002; *w*: *b* = 0.0005, *p* = 0.79, *R*^2^ = 0.0004). Thus, there is no sign of learning in either group, and in the feedback condition the sign of b is even negative for PC and positive for *w*, suggesting, if anything, a deterioration rather than an improvement of performance. There is a possibility that learning effects are so rapid that they would occur even within the first 100 trials of learning and thus not be detectable in Figure 1. Although this seems a priori implausible we undertook an even more fine-grained analysis of data from the first learning block. We found no effects on PC [with 10 trials the fitting of *w* is unfeasible (Mazzocco et al., 2011)] indicating the presence of such extremely rapid learning with the same analyses undertaken on this subset of data (feedback: *b* = 0.001, *p* = 0.68, *R*^2^ = 0.00; control: *b* = 0.00, *p* = 0.95, *R*^2^ = 0.00, with the first learning block divided into sub-blocks of 10 trials).

The results suggest an effect of feedback on motivation rather than on learning. The correlation between motivation and overall performance after feedback was introduced was significant both for PC [*r*_(37)_ = 0.37, *p* = 0.019] and for *w* [*r*_(37)_ = −0.40, *p* = 0.012], confirming this suspicion. A One-Way ANOVA revealed that feedback had a strong effect on self-rated motivation, which was higher in the feedback group (*M* = 4.2, *SD* = 0.4) than in the control group (*M* = 3.2, *SD* = 0.8), [*F*_(1, 37)_ = 20, *p* < 0.001]. The average performance on all trials after introduction of feedback is significantly better in the feedback condition (PC: *M* = 0.78, *SD* = 0.03; *w*: *M* = 0.20, *SD* = 0.05) than in the control condition (PC: *M* = 0.76, *SD* = 0.03; *w*: M = 0.24, *SD* = 0.06) [PC: *F*_(1, 37)_ = 4.2, *p* = 0.047; *w*: *F*_(1, 37)_ = 4.2, *p* = 0.048]. We conducted a GLM ANCOVA on PC, with motivation as covariate and test part (pre-test, training, post-test) and condition (control/feedback) as independent variables, to investigate participants' performance in the two conditions when equating their level of motivation. The GLM ANCOVA adjusts the means in the two conditions with respect to motivation and adjusts the independent variable for interactions with the covariate. The adjusted means in the control (*M* = 0.762, *SEM* = 0.009) and feedback (*M* = 0.764, *SEM* = 0.009) conditions were very similar and the effect of feedback was not significant [*F*_(1, 36)_ < 1]. We interpret this finding to signify that feedback has little or no effect on performance above an indirect effect through motivation (The size of this motivational effect in terms of PC is modest; feedback condition, *Mdn PC* = 0.79, [0.69 – 0.86]; control condition *Mdn PC* = 0.77, [0.64 – 0.87].).

To control for the use of cumulative area/average dot-size as perceptual cues, half of the trials were cumulative area controlled whereas the other half was controlled for dot-size. To establish whether participants use these cues, a *P*_cue_ *index* was calculated by subtracting performance on trials where cumulative area was controlled for from performance on trials where average dot-size was held constant. This index (presented in Table 1) was close to zero before, during, and after onset of feedback and not significantly different between the control and feedback group at any test. It is possible that participants are heterogeneous, some using cumulative area as a cue whereas others rely on size. This will lead to some participants scoring better for area controlled stimuli and others scoring better for size-controlled stimuli, and would turn up as an increase in variance of the differences of the scores for these both item-types (i.e., analogous to the “rectified index” used by DeWind and Brannon). To test for this possibility we compared the variances of the difference in PC for the two item-types (area/size controlled) between the control group and the experimental group with *F*-ratio variance tests. Variances were highly comparable throughout the entire training phase and when comparing the conditions no *F*-ratio test revealed a statistically significant effect. Thus, although we cannot fully exclude the possibility that participants used perceptual cues, we have no indications that they did.

The correlation between accuracy in the inspection time task and overall performance in the ANS task, when controlling for motivation, [PC: *r*_(36)_ = 0.39, *p* = 0.02; *w*: *r*_(36)_ = −0.47, *p* = 0.003] was significant, with better performance in the inspection time task related to better ANS acuity (higher PC/lower *w*). In the previous correlation and those reported below motivation was partialled out because motivation had an effect on PC and *w*. To investigate to what degree participants had insight in their performance we correlated estimated percentile rank with actual percentile rank for both the ANS acuity task (both *w* and PC) and the inspection time task. Neither the correlation for ANS acuity [PC: *r*_(37)_ = 0.23, *p* = 0.17; *w*: *r*_(37)_ = 0.18, *p* = 0.27] nor inspection time [*r*_(36)_ = 0.05, *p* = 0.77] was significant (partial correlations controlling for motivation in both tests). For ANS acuity, participants rated themselves slightly, but not significantly, below the median [49th percentile: *t*_(38)_ = 0.18, *p* = 0.86]. For inspection time, the average rated percentile was 33. This underestimation of the participants' own performance was statistically significant [*t*_(37)_ = 3.2, *p* = 0.003].

## Discussion

Recent research on children (e.g., Wilson et al., 2006b, 2009; Mazzocco et al., 2011) and adults (Tokita and Ishiguchi, 2010; DeWind and Brannon, 2012) has raised the question whether it is possible to improve ANS acuity by training. Specifically, the results in DeWind and Brannon (2012) suggest that we should expect effects on ANS acuity to occur rapidly when feedback is introduced. However, the lack of control groups, and other features in the designs, makes it difficult to separate effects on ANS acuity per se from task practice effects, perceptual learning, or motivational effects.

In the present study we investigated the cause of the rapid effects of feedback on acuity in the ANS using a control group design and controlling for strong perceptual effects. To account for motivational effects we also obtained self-rated motivation scores. Our results showed a small advantage for the feedback group, but no signs of incremental learning, which suggests the operation of a motivational effect of *introducing* feedback rather than a *function of* the training with feedback. To our knowledge, no previous study has shown an effect of motivation on ANS acuity.

The motivational effect is probably due to the monotonous task becoming more interesting for participants, when they can monitor their own performance. It should be noted, though, that performance in the control condition was stable, indicating that participants were impressively resistant to fatigue and boredom. The similarly stable performance in the feedback condition over the 10 blocks of training also indicates that the lack of a learning effect is not due to participants becoming fatigued. The ANS is considered a core cognitive system in which representations of numerosity are formed automatically (see e.g., Gallistel and Gelman, 2000). As such it is reasonable to assume that motivation mainly plays a role before (e.g., attention during visual processing) or after (e.g., when the two representations are compared) the representations of the two sets are formed. While the present study was not designed to answer the question of at what stage motivational effects enter, it is certainly an interesting one for future research. It should be noted that participants in the DeWind and Brannon study were paid for each correct response. This procedure would probably reduce an effect of motivation. However, because all participants received the monetary incentive and because no measure of motivation was collected any conclusions as to the influence of motivation on their subjects are difficult to make.

The participants in DeWind and Brannon (2012), on average, improved their *w* from 0.43 to 0.30 when feedback was introduced. Previous results indicate that *w*, for adults, is normally found in the interval 0.1 – 0.45 with a median in the order of 0.25 (Pica et al., 2004; Halberda and Feigenson, 2008; Halberda et al., 2008; Tokita and Ishiguchi, 2010). Data collected in our own lab from almost 200 participants (undergraduate students), using the task based on Halberda et al. (2008) described above, indicate a population median *w* of 0.21 [0.11 – 0.43]. Compared to our data, participants in the study by DeWind and Brannon (2012) started out with an average in the lowest (poorest) 1st percentile before training and moved upwards to the lowest 11th percentile after training. Thus, whatever participants in that study may have learned, they did not show signs of exceptional ANS acuity after training, but performed in fact at very low levels compared to other studies. Undoubtedly, differences in stimuli may account for some of this performance discrepancy and some previous studies (Price et al., 2012) indicate that comparisons of absolute levels of *w* between tasks and studies should be made with caution. However, given the similarity of tasks, with simultaneously presented spatially intermixed dots and the same exposure time performance could be expected to be more comparable. Rather than signaling an impressive effect of feedback on ANS acuity this seems to indicate that participants may have learned to ignore misleading perceptual cues to achieve the improvement in numerosity discrimination.

While it is important for researchers to be aware of perceptual cues, we fear that exaggerated measures to minimize their use may be counterproductive. More specifically, creating stimuli in a Stroop-like fashion with perfect positive (congruent stimuli) or perfect negative (incongruent stimuli) correlation between a perceptual cue and numerosity possibly makes perceptual cues more salient. This, in turn, may induce a large number of participants, who otherwise would not have done so, to rely on them (see e.g., Inglis et al., 2011 where a substantial proportion of participants had to be excluded from the analysis due to suspected use of perceptual cues). Undoubtedly, if participants are encouraged to rely on perceptual cues they can learn to do so. The procedure of creating congruent and incongruent stimuli should not be confused with a control procedure where each trial controls for a separate perceptual cue. Such a procedure makes the correlation between a specific cue and numerosity zero on controlled trials and well below one on uncontrolled trials. In effect, the control procedure makes the controlled for perceptual cues very poor predictors for numerosity.

The correlations between performance in the inspection time task and the ANS acuity task suggest that ANS acuity is possibly related to the more general ability to quickly perceive and discriminate between perceptual stimuli. Several studies have shown correlations between inspection time and psychometric intelligence (see e.g., Deary and Stough, 1996), which opens for the possibility that the ANS-Inspection time correlation alternatively may be due to ANS being more generally related to cognitive functioning, rather than the more specific ability it has been thought to be^6^. The significant ANS-Inspection time relationship reported here is in contrast to the findings documented by Halberda et al. (2008) who found that neither performance in a rapid automatic naming task (R.A.N., Denckla and Rudel, 1976) nor visual working memory accounted for a significant proportion of variance in ANS acuity. However, while the R.A.N.-task requires participants to access word labels for a presented color, and thus is not merely a perceptual task, the inspection time task only requires a rapidly formed perceptual representation of the difference between two stimuli. The difference in results may thus be due to differences in task demands, a possibility that should be explored in future research. DeWind and Brannon (2012) included a line length task and reported a significant positive correlation between *w* for number discrimination and *w* for line length discrimination. This indicates that discrimination of different magnitudes could be carried out by a common system (see also, Cohen Kadosh et al., 2008). While the line length task in DeWind and Brannon (2012) and our inspection time task are similar and both include lines as stimuli there are non-trivial differences between the tasks. In the inspection time task the perceptual difference between the stimuli is highly discriminable if participants would have unlimited presentation time, whereas this may not be the case for some stimuli in the line length task. Thus, while the line length task probably to a certain degree measures perceptual discrimination ability the inspection time task is purported to measure “pure” mental processing speed. Therefore, the correlation reported by DeWind and Brannon (2012) and the correlation reported here are possibly indicative of two separate relationships between ANS and other abilities. Further, our results suggest that some of the observed relationship between ANS acuity and general math achievement (e.g., Halberda et al., 2008; Mazzocco et al., 2011) may be mediated by motivation, those more motivated in the numerosity judgments may also have been more motivated to perform well at math. The effect of motivation on ANS acuity is small, however.

The lack of significant correlation between self-rated and actual performance suggests that ANS acuity is a core ability that lies beyond meta-cognitive monitoring^7^. The strong underestimation of participants' performance in the inspection time task reveals that they find this task very hard.

Most importantly, the results of the present study show that training on non-symbolic tasks alone does not seem in the short-term to improve the number sense rapidly. This, in turn, suggests that a characteristic of the number sense may be its inertia rather than malleability, at least in adults. It remains possible, of course, that more sustained training may result in learning. Further, it remains possible that a critical period exists when practice may lead to improvement. It remains to be investigated if the effects of training found in children (Wilson et al., 2006b, 2009) can be extended to tasks only including non-symbolic comparisons and when control groups are used. Finally, it is also possible that ANS acuity could be improved by other means than corrective feedback on magnitude comparison judgments. For example, using feedback on symbolic labeling of non-symbolic stimuli might improve the mapping between these kinds of representations. Future research should explore these possibilities.

### Conflict of interest statement

The authors declare that the research was conducted in the absence of any commercial or financial relationships that could be construed as a potential conflict of interest.

## Footnotes

^1^While at least in theory one cannot exclude the possibility that the ANS may sharpen spontaneously in the absence of feedback, we find this unlikely and we know of no study that has reported such a phenomenon. Humans probably engage in activities that activate the ANS on a daily basis. If the mere use of the system would lead to its refinement, there would thus seem to be ample opportunity for high level performance that probably would leave no room for further improvement by laboratory training.

^2^We are most grateful to Nicholas DeWind for providing the original data that allowed for these analyses.

^3^In limiting our study to this amount of feedback, we of course acknowledge that more substantial training might have effects above what is found in this study.

^4^DeWind and Brannon used a prolonged training period spread out over days. However, all effects where obtained during the first feedback session which occurred at one single occasion. Thus, it is not possible that their results are due to effects of feedback that are measurable only after a consolidation (i.e., overnight) period that would not turn up in the present study.

^5^One participant in the feedback condition who performed at chance levels was excluded. An additional participant performing below chance levels in the inspection time task was excluded from analyses involving inspection time.

^6^Another possibility is that ANS acuity and performance on the inspection time task are related through a common representation of magnitude, as suggested by Walsh (2003) in a theory of magnitude (ATOM). See also Cohen Kadosh et al. (2008).

^7^Studies of realism of metacognitive judgments (often focused on the so called overconfidence phenomenon) have generally made a distinction between three kinds of such judgments (see Merkle and Weber, 2011); judgments of one's absolute performance, confidence in the precision of one's estimates, and judgements of one's skill relative to others (e.g., the “better than average effect”). In the present study we were interested in the latter kind of relative judgements. It is however, possible that participants were able to monitor their performance on a trial-by-trial basis.
